# Do Preoperative Vastus Medialis Volume and Quality Affect Functional Outcomes after Total Knee Arthroplasty?

**DOI:** 10.3390/jcm11133618

**Published:** 2022-06-23

**Authors:** Ho Jung Jung, Min Wook Kang, Jong Hwa Lee, Joong Il Kim

**Affiliations:** Department of Orthopaedic Surgery, Kangnam Sacred Heart Hospital, Hallym University College of Medicine, 1 Singil-ro, Yeongdeungpo-gu, Seoul 07441, Korea; hodge.jung@gmail.com (H.J.J.); kmwtop@naver.com (M.W.K.); bigdawg@hallym.or.kr (J.H.L.)

**Keywords:** vastus medialis, volume, fat infiltration, functional outcomes, total knee arthroplasty

## Abstract

Background: Vastus medialis function has been implicated in the development and progression of knee osteoarthritis; however, studies evaluating the influence of its preoperative volume and quality on functional outcomes following total knee arthroplasty (TKA) remain scarce. This study aimed to determine the association between vastus medialis volume, quality, and clinical outcomes after TKA. Methods: Among the patients who underwent unilateral TKA, 92 who had undergone magnetic resonance imaging (MRI) before TKA were included. Preoperative vastus medialis volume and quality were assessed using the cross-sectional area (CSA) and fat infiltration ratio on MRI. Clinical outcomes were evaluated using the Knee Society knee score (KSKS) and Knee Society function score (KSFS) at baseline and 1 year after surgery. The relationships between preoperative CSA, fatty infiltration ratio, and clinical outcomes were analyzed using univariate and multivariate linear regression. Results: Only the fatty infiltration ratio was significantly associated with postoperative KSFS in the univariate linear regression analysis. In the multivariate linear regression analysis, age and fatty infiltration ratio were significantly associated with postoperative KSFS. Conclusions: Increased vastus medialis fat infiltration is associated with worse functional outcomes after TKA. Preserving vastus medialis quality could improve functional outcomes, and surgeons should encourage patients to perform quadriceps strengthening exercises before surgery.

## 1. Introduction

Patients with severe knee osteoarthritis (OA) who undergo total knee arthroplasty (TKA) commonly experience quadriceps muscle weakness owing to disuse atrophy secondary to pain in the involved knee joint [1,2]. After TKA, patients usually experience quadriceps muscle weakness of 50–60% of its preoperative level, and it is unclear whether quadriceps muscle strength can be fully recovered by postoperative rehabilitation [3]. Several reports have shown that patients who underwent TKA could present functional disabilities related to poor quadriceps muscle function for long periods after TKA [4,5,6]. Despite the importance of quadriceps muscle function, little is known about the association between the preoperative quadriceps muscle status and the postoperative clinical outcomes after TKA.

The vastus medialis (VM) is a part of the quadriceps muscle considered to be the most crucial part of quadriceps muscle function [7]. Recently, the VM volume has been suggested to be associated with poor functional outcomes after TKA [8]. However, the muscle volume may not be solely responsible for muscle status and several studies have shown that muscle function is poorly correlated with muscle volume loss [9,10]. Instead, changes of muscle composition characterized by muscular fibrosis and a resultant intramuscular fat infiltration should be considered. Several studies have suggested that an increase in intramuscular fat is associated with the loss of muscle function with respect to strength and mobility [11,12]. Therefore, compared to reduction of volume, increased intramuscular fat infiltration may be a better predictor of muscle function [10]. Nevertheless, there are limited studies evaluating the influence of preoperative VM fat infiltration on functional outcomes after TKA. To this end, this study aimed to determine the association between the preoperative VM volume, fat infiltration, and clinical outcomes after TKA. Our hypothesis is that preoperative VM fat infiltration, rather than VM volume, may be associated with postoperative clinical outcomes after TKA.

## 2. Materials and Methods

### 2.1. Patients

**Figure 1:**
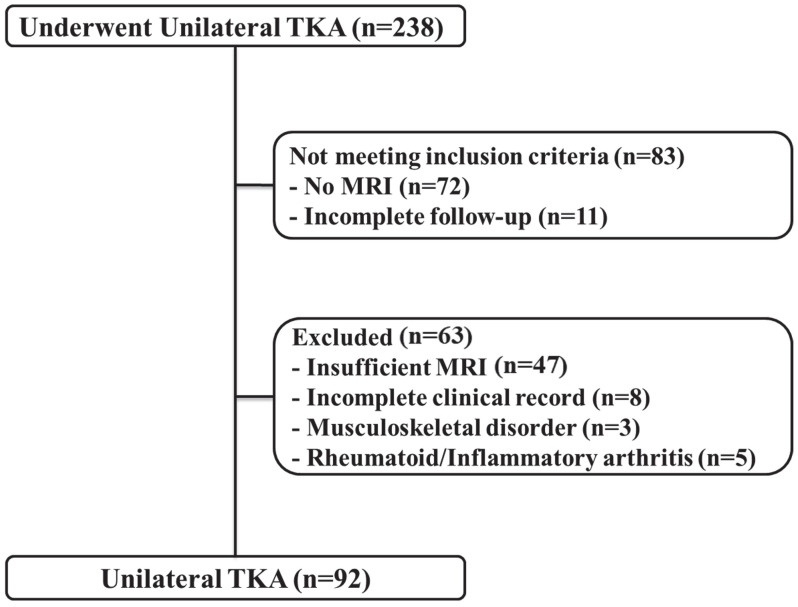
Flow diagram for the patients’ enrollment.

We retrospectively reviewed the medical records of patients who underwent unilateral TKA between July 2019 and February 2021. Inclusion of patients was narrowed down to those who had been subjected to knee magnetic resonance imaging (MRI) before surgery and had a complete postoperative follow-up record 1 year after surgery. Exclusion criteria were: (1) insufficient MRI data that did not cover the distal 10% of the femoral length; (2) incomplete clinical records; (3) the presence of other musculoskeletal disorders that affect the postoperative rehabilitation program; and (4) the presence of rheumatoid or inflammatory arthritis. Finally, 92 patients were included in the study (Figure 1). The demographic characteristics of the study population are shown in Table 1. This study was approved by the institutional review board of our hospital (2021-11-030).

### 2.2. Surgical Technique and Rehabilitation

All the patients underwent primary unilateral TKA using the same surgical protocol. Using a standard medial parapatellar approach and tourniquet inflation (300 mmHg), a posterior stabilizing prosthetic (Persona^®^, Zimmer, Warsaw, IN, USA) was implanted with patellar resurfacing. The tourniquet was deflated after the final fixation of the cemented prosthesis, and the remaining bleeding focus was cauterized after manual compression through gauze packing on the surgical site during cement hardening. A closed suction drain was placed in the joint and capsule closure was performed in a watertight fashion. Range of motion (ROM) exercises were initiated on postoperative day (POD) 1. The drain was removed and ambulation with a walker was initiated on POD 2.

### 2.3. Radiographic Evaluation

**Figure 2:**
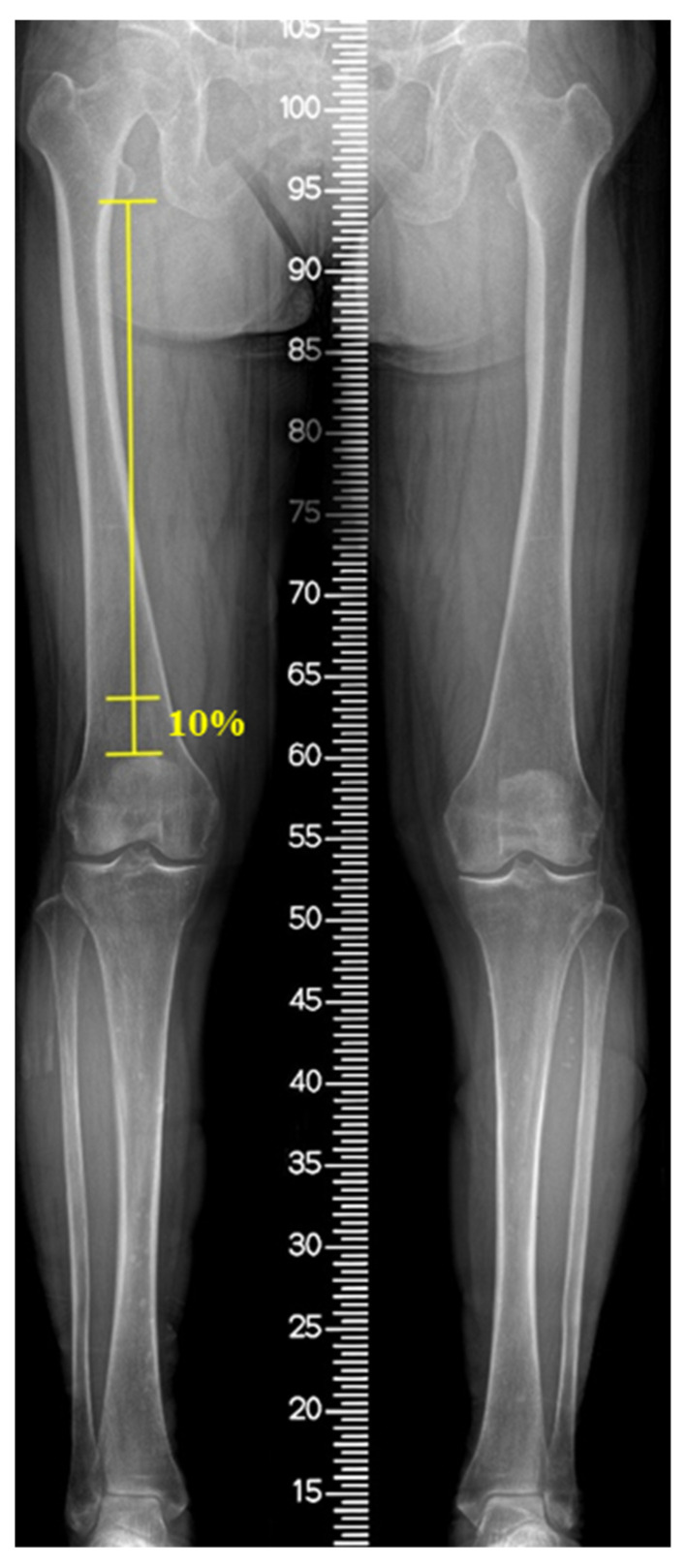
Measurement of the femoral length (FL), defined as the distance from the distal end of lesser trochanter to the superior surface of patella in full-length standing anteroposterior radiograph.

**Figure 3:**
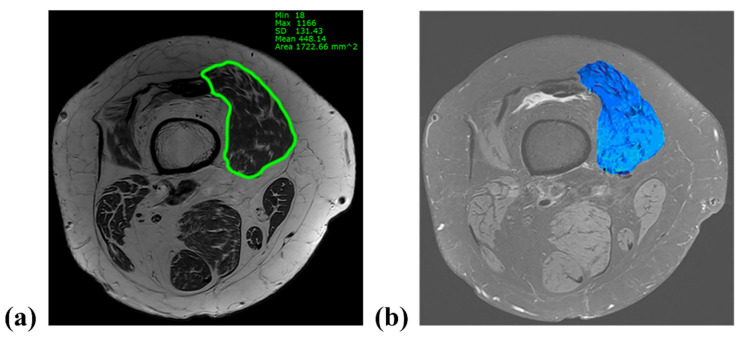
Measurement of cross-sectional area (CSA) and fat infiltration (FI) ratio on axial magnetic resonance imaging. (**a**) The vastus medialis (VM) CSA was acquired from the axial proton density image after marking the VM margin using the embedded tools of the picture archiving communication system software. (**b**) The area corresponding to the VM was color-mapped with 16 different colors for each signal. Color pixels derived from the fat signal were extracted, and the FI ratio was defined as the percentage of the number of fat pixels to the sum of the VM pixels.

Patients underwent MRI (Magnetom Skyra; Siemens Medical Solutions, Erlangen, Germany) with the knee fully extended in the supine position and stabilized by a knee holder. During MRI, the knee joint (between 1/3rd of the distal femur and 1/3rd of the proximal tibia) was fully covered and contained axial slices of proton density (PD) and PD fat suppression at 2 mm intervals. The VM cross-sectional area (CSA) and the fat infiltration (FI) ratio were measured at the distal 10% level of the femoral length. This level has been previously reported to reflect the total quadriceps volume in patients with knee OA [13]. The femoral length was defined as the length from the distal end of the lesser trochanter to the superior surface of the patella on a full-length standing anteroposterior radiograph (Figure 2). On coronal MRI view, the level was identified from the superior surface of the patella and the corresponding axial slice was matched using 3D indicators of the picture archiving communication system (PACS; M6, Infinitt Healthcare, Republic of Korea). The VM CSA was acquired from the axial PD image after marking the VM margin using the embedded tools of the PACS software. To assess the FI ratio of the VM, the marked area on the PD slice was transferred to the corresponding PD fat-suppression slice and the intramuscular fat signal within the area was confirmed by comparing the PD and PD fat-suppression slices. Thereafter, the area was color-mapped using 16 different colors for each signal. Color pixels corresponding to the signal of the fat tissue were extracted, and the FI ratio was defined as the percentage of the pixels of the fat tissue to the sum of the pixels of VM (Figure 3). The hip–knee–ankle (H–K–A) angle was determined as the angle formed by the mechanical axis of the femur and the tibia on full-length standing anteroposterior (AP) radiographs. To classify the severity of OA, the tibiofemoral joint was evaluated using the Kellgren–Lawrence scale in standing AP and lateral radiographs as follows: grade I, doubtful narrowing of the joint space and possible osteophytic lipping; grade II, definite osteophytes and possible narrowing of the joint space; grade III, moderate multiple osteophytes and definite narrowing of the joint space; and grade IV, large osteophytes-marked narrowing of the joint space [14].

### 2.4. Clinical Evaluation

Clinical evaluation was performed by a single clinical investigator, to prevent bias, using the Knee Society knee score (KSKS) and Knee Society function score (KSFS) before surgery and 1 year after surgery. The operative time was determined as the time from skin incision to wound closure, as confirmed by the anesthesiologist.

### 2.5. Statistical Analysis

The associations between the VM CSA/FI and clinical scores were analyzed using univariate and multivariate linear regression analyses. Radiographic parameters were measured twice by two independent observers, with a 2-week interval between measurements. Intra- and inter-observer measurement reliabilities were assessed using the intraclass correlation coefficient (ICC). All statistical analyses were performed using SPSS Statistics (version 27.0, IBM, USA). Statistical significance was set to *p* < 0.05.

## 3. Results

**Figure 4:**
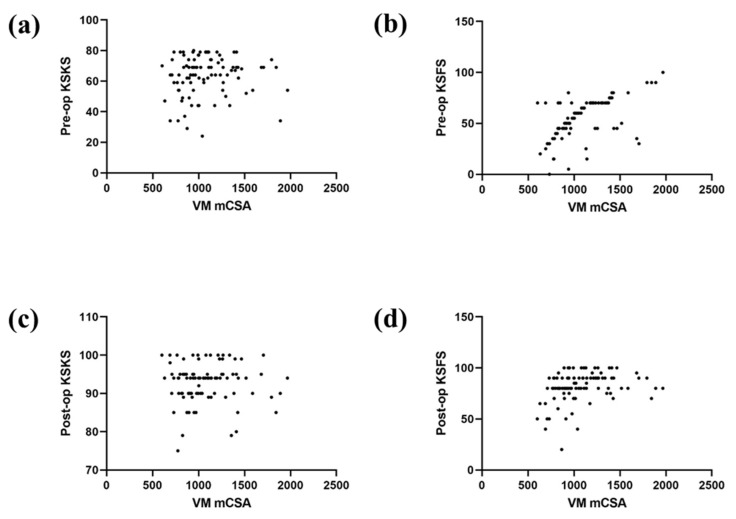
Scatter plots of the univariate relationship between CSA and (**a**) preoperative Knee Society knee score (KSKS); (**b**) preoperative Knee Society functional score (KSFS); (**c**) postoperative KSKS; (**d**) and postoperative KSFS.

**Figure 5:**
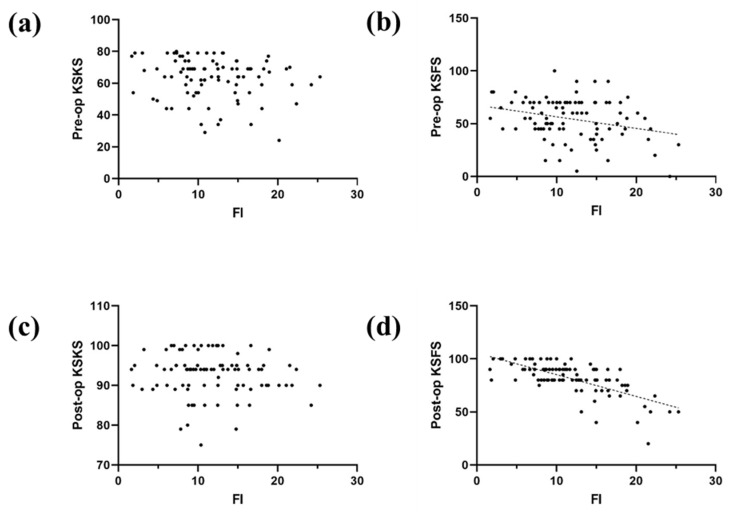
Scatter plots of the univariate relationship between FI ratio and (**a**) preoperative KSKS; (**b**) preoperative KSFS; (**c**) postoperative KSKS; (**d**) and postoperative KSFS.

The mean preoperative VM CSA and FI ratio were 1083.4 ± 296.2 mm^2^ and 11.8 ± 5.1%, respectively. Univariate linear regression analysis identified the preoperative FI ratio to be associated with preoperative KSFS (β = −1.109; *p* = 0.005) but not with preoperative KSKS (*p* = 0.104). Preoperative VM CSA was not associated with preoperative KSKS (*p* = 0.628) or KSFS (*p* = 0.323). Although the preoperative VM CSA was not associated with postoperative KSFS (*p* = 0.323), the preoperative VM FI ratio was significantly associated with the postoperative KSFS (β = −2.164; *p* < 0.001). The preoperative VM CSA (*p* = 0.431) and FI ratio (*p* = 0.376) were not associated with the postoperative KSKS (Figure 4 and Figure 5). Multivariate linear regression analysis, including confounding factors, identified age (β = −0.533; *p* = 0.020) and the VM FI ratio (β = −1.744; *p* < 0.001) to be significantly associated with postoperative KSFS (Table 2). The intra- and inter-observer measurement reliabilities were identified to be excellent for all parameters (ICC > 0.8, range: 0.82–0.91).

## 4. Discussion

In our study, we found that the preoperative VM volume and fat infiltration ratio were not associated with the postoperative KSKS. However, we established that the preoperative VM fat infiltration ratio was significantly associated with the postoperative KSFS, although the preoperative VM volume was not significantly associated with the postoperative KSFS. To our knowledge, this is the first clinical study to investigate the effects of the preoperative VM intramuscular fat infiltration on functional outcomes after TKA.

The quadriceps muscle plays a crucial role in knee extension and is the main active knee stabilizer. After TKA, the quadriceps muscle strength is a major determinant of physical function and is associated with the return to daily activities [15]. Some studies suggested that the preoperative quadriceps muscle status could affect the postoperative outcomes after TKA [16,17,18]. Mizner et al. [16] arrived at this conclusion by analyzing the relationship between preoperative quadriceps muscle strength and the 1-year postoperative timed up-and-go test/stair climbing test. Zeni et al. [17] also found that weak preoperative quadriceps strength increased handrail use indicating the patients’ difficulty in climbing stairs 2 years after TKA. In another prospective cohort study, Christensen et al. [18] showed that, although no other factors were relevant, the preoperative quadriceps muscle strength index was the only predictive factor positively associated with postoperative mobility tasks. Similarly, our study confirmed that preoperative quadriceps muscle status could affect the postoperative functional outcomes after TKA. However, no study has focused on the quality of the quadriceps muscle, and we believe that our study paves the way for useful clinical implications, as it is the first to analyze the relationship between the quality of the quadriceps muscle and functional outcomes after TKA.

In this study, the VM fat infiltration ratio was significantly associated with postoperative functional outcomes rather than with the VM volume. Several studies have reported that muscle quality such as intramuscular fat infiltration is more important than muscle volume for muscle function [19,20,21]. Kumar et al. [19] showed that higher quadriceps intramuscular fat fractions, but not volume, worsened the knee injury and osteoarthritis outcome score by multiple linear regression analysis. In addition, Goodpaster et al. [20] reported that intramuscular fat values on CT were associated with lower muscle strength in older patients and that muscle strength was independent of muscle quantity. Through a comparative study, Inhuber et al. [21] analyzed the effect of the quadriceps muscle fat fraction and CSA on the isometric contraction force. The study found that the fat fraction of the quadriceps muscle, but not the CSA, was a significant predictor of quadriceps muscle strength in multivariate regression models.

We evaluated the VM volume and fat infiltration ratio at the distal 10% of femoral length using routine knee MRI rather than thigh MRI. Because routine knee MRI involves only a few slices of the distal femur, the entire VM cannot be evaluated. However, many studies have shown that a single axial slice at the proper level of the femur can predict the status of the entire thigh muscle using computed tomography (CT) or MRI [13,22,23]. Morse et al. [22] reported that the total quadriceps muscle volume can be estimated from a single CSA of the quadriceps muscle at 60% from the distal end of the muscle length in healthy adults. Marcon et al. [23] also reported that the CSA of the quadriceps muscle at the mid-thigh level reflects the total quadriceps muscle volume in patients who underwent anterior cruciate ligament reconstruction. However, elderly patients with knee OA usually had muscle belly atrophy, especially in the mid-thigh, and a different estimation method was required. Yamauchi et al. [13] suggested the novel finding that a CSA of VM at the distal 10% level of the femoral length was the best predictor of the overall VM volume in patients with knee OA. In addition, Kim et al. [8] and Mersmann et al. [24] showed that the level showing the maximal CSA of VM was 20% proximal to the most distal end of the lateral femoral condyle, which was similar to our measurement level. Regarding the imaging options for evaluation, CT bears limitations such as radiation exposure, making it difficult to distinguish between intra- and inter-muscular fat [25,26]. Although MRI is time consuming and high in cost, it clearly distinguishes fat and muscle by their different signal intensities [27,28]. In this study, MRI was chosen to accurately classify the fat component by comparing the overall PD and the PD fat suppression images. Therefore, we assume that our measurement protocols with MRI can sufficiently reflect the VM status in terms of both quality and quantity.

Our study highlighted the importance of preoperative quadriceps muscle strengthening exercises before TKA because preoperative VM status was associated with functional outcomes. Along these lines, several studies have shown that preoperative quadriceps muscle exercise could lead to better clinical outcomes by improving the quadriceps muscle strength and eventually to an improved quality of life after TKA [29,30,31,32,33]. An et al. [29] reported that patients who participated in a 3-week intensive exercise program before bilateral TKA demonstrated better clinical results in the Western Ontario and McMaster Universities Osteoarthritis (WOMAC) index than the usual treatment group 1 month after TKA. In the same manner, Tungtrongjit et al. [30] found that patients who participated in a preoperative quadriceps muscle strengthening exercise program for 3 weeks experienced significantly lower pain 3 months postoperatively (61.5% compared to control group) and WOMAC scores of 70.1% and 63.8% at 1 and 3 months after surgery, respectively. Another systematic review analyzing 131 studies reported that preoperative quadriceps muscle exercises can lead to better functional outcomes in stair climbing test, 30-s chair rise test, and 6-min walk test after TKA within 2 years postoperatively [31]. Therefore, surgeons should thoroughly evaluate quadriceps muscle function before TKA and encourage patients to perform quadriceps muscle strengthening exercises to achieve better functional outcomes, particularly those with poor preoperative functional scores.

Our study has a few limitations. First, it was performed using a relatively small sample size and bore a retrospective design. Thus, further well-designed prospective investigation is needed to support our findings. Second, we only evaluated the VM status among the quadriceps muscles. However, numerous studies have found that VM performs the main function during ambulation among quadriceps muscle groups and that VM impairment increases joint reaction force and leads to potential complication after TKA [7,34,35]. We believe that the VM alone could reflect the state of the entire quadriceps muscle.

## 5. Conclusions

The preoperative VM fat infiltration ratio was associated with worse functional outcomes after TKA, while the preoperative VM volume was not. Preservation of VM quality may have the potential to improve functional outcomes after TKA; thus, orthopedic surgeons should encourage patients to perform quadriceps muscle strengthening exercises before TKA to maintain VM quality.

## Figures and Tables

**Table 1:** Patient characteristics.

	Group (*n* = 92)
Age (years) *	70.4 ± 7.7
Sex (male:female)	18:74
BMI (kg/m^2^) *	26.3 ± 4.0
K–L grade III–IV	30:62
H–K–A angle (°) *^,†^	8.3 ± 4.8
Operation duration (min) *	92.6 ± 12.7

* Values are presented as the mean ± standard deviation. ^†^ Positive value indicates varus alignment and negative value indicates valgus alignment. BMI, body mass index; K–L, Kellgren–Lawrence; H–K–A, hip–knee–ankle.

**Table 2:** Multivariate linear regression analysis of variables associated with postoperative KSFS.

Variables	Postoperative KSFS	
	β Coefficient (SE)	*p*-Value *
Age (years)	−0.533 (0.177)	**0.020**
$\mathrm{BMI}\;(\mathrm{kg}/m^{2}$)	−0.319 (0.275)	0.304
FI ratio (%)	−1.744 (0.262)	**<0.001**
H–K–A angle (deg)	−0.533 (0.211)	0.817
Operation time (min)	−0.218 (0.076)	0.190

* Statistically significant *p*-values are shown in bold. KSFS, Knee Society functional score; BMI, body mass index; FI, fat infiltration; H–K–A, hip–knee–ankle.

## Data Availability

The data presented in this study are available on request from the corresponding author. The data are not publicly available.
